# 

**Published:** 1874-05

**Authors:** 


CONTRIBUTIONS TO THE STUDY OF YELLOW FEVER, by J. M. Toner, M.D., and John M. Woodworth, M.D., re-printed from the annual report Supervising Surgeon United States Marine Hospital Service, 1873. Washington, 1874. Octavo; paper, pp. 51.

---

EXTRACT FROM A REPORT ON THE HISTORY OF THE SURGERY OF TENNESSEE, COMPRISING THE SUBJECTS OF STONE IN THE BLADDER, OVARIAN TUMORS AND VESICO-VAGINAL FISTULA; MADE TO THE TENNESSEE STATE MEDICAL SOCIETY, APRIL 3, 1872, by Wm. T. Briggs, M.D., Professor of the Principles and Practice of Surgery in the Medical Department of the University of Nashville. Nashville, Tenn., 1874. Reprint from the Nashville Journal of Medicine and Surgery. Octavo, paper, pp. 98. Illustrated by Photograph.

Medical and Pharmaceutical Notes on the Preservation of Hypodermic Solutions, by Edward H. Squibb; on Ergot and its preparations; on Rhubarb; on Physicians Pocket Cases; on a General Apparatus Stand, ect., by Edward R. Squibb, M.D., of Brooklyn, New York. Philadelphia, Sherman & Co., Printers, 1874. Octavo; paper, pp. 66.

---

Esmarch's Bloodless Operation on the Extremities, with Cases, by Paul F. Eve, M.D. Nashville, Tenn. Prepared for State Medical Society. Pamphlet, pp. 6.

---

Writers' Cramp, or Scrivners' Palsy, by Reuben A. Vance, M.D., New York. Reprint from Boston Medical and Surgical Journal, Boston. David Clapp & Son, 1873. Octavo, pp. 12.

---

Skin Grafting, by R. J. Levis, M.D., Surgeon to the Pennsylvania Hospital and to the Wills Ophthalmic Hospital. Reprint from the Philadelphia Medical Times. Octavo, paper, pp. 8.

---

Herpes Gestationis, a Rare Affection of the Skin, Peculiar to Pregnancy, by L. Duncan Bulkley, M.D., New York. Willliam Wood & Co., 1874. Octavo, paper, pp. 32.

---

Treatise on the Hot Springs of Arkansas, Dr. A. S. Garnett. Octavo, paper, pp. 48.

---

Intorno L'Fficia Particolarmente Anticoerica del Solfuro Nero di Mercurio detto comunemente Etiope Minerale, discorso dettato per l'xi Congresso degli Scienziati Italiani, dal dottor Socrate Cadet e cenno del come il signor Colonnello Alessandro Calandrelli, si liberasse in Vienna da non lieve assalto indocolerico. Roma: Stablimento Typograpico di G. Via Corso 387.

### ACKNOWLEDGMENTS.

Atlanta Daily Herald; American Journal of Pharmacy; American Practitioner; American Journal of Medical Sciences; Archives of American Medicine and Surgery; Baltimore Physician and Surgeon; Boston Medical and Surgical Journal; The Clinic; Chicago Med. Journal; Cincinnati Lancet and Observer; Detroit Review of Medicine and Pharmacy; Druggists' Circular; The Galaxy; Herald of Health; Journal of Chemistry; Journal of Materia Medica; Medical and Surgical Reporter; Medical Record; Medical Examiner; Medical News and Library; The Meteor (Ala. Insane Hospital); Nashville Medical and Surgical Journal; New York Medical Journal; Northwestern Medical and Surgical Journal; Pacific Medical and Surgical Journal; Philadelphia Medical Times; Richmond and Louisville Med. Journal; Rome Daily Commercial; St. Louis Medical and Surgical Journal.

